# Endovascular embolization techniques in acute thoracic and abdominal bleedings can be technically reproduced and trained in a standardized simulation setting using SLA 3D printing: a 1-year single-center study

**DOI:** 10.1186/s13244-022-01206-7

**Published:** 2022-04-09

**Authors:** Reinhard Kaufmann, Christoph J Zech, Michael Deutschmann, Bernhard Scharinger, Stefan Hecht, Klaus Hergan, Richard Rezar, Wolfgang Hitzl, Matthias Meissnitzer

**Affiliations:** 1 Department of Radiology, University Hospital Salzburg, Paracelsus Medical University, Müllner Hauptstraße 48, 5020 Salzburg, Austria; 2 Clinic of Radiology and Nuclear Medicine, University Hospital Basel, University of Basel, 4031 Basel, Switzerland; 3 Clinic of Internal Medicine II, Department of Cardiology and Internal Intensive Care Medicine, University Hospital Salzburg, Paracelsus Medical University, 5020 Salzburg, Austria; 4 Research Office (Biostatistics), Paracelsus Medical University of Salzburg, 5020 Salzburg, Austria

**Keywords:** Embolization, Simulation, 3D Printing, Arterial bleedings, Coiling

## Abstract

**Background:**

Endovascular embolization techniques are nowadays well established in the management of acute arterial bleedings. However, the education and training of the next generation of interventionalists are still based on the traditional apprenticeship model, where the trainee learns and practices directly at the patient, which potentially affects the patient’s safety. The objective of this study was to design and develop a standardized endovascular simulation concept for the training of acute bleeding embolizations, based on real-life cases.

**Results:**

An adaptable and cost-effective endovascular simulator was developed using an in-house 3D print laboratory. All thoracic and abdominal acute bleeding embolizations over more than a year with appropriate pre-interventional computed tomography scans were included to manufacture 3D printed vascular models. A peristaltic pump was used to generate pulsatile flow curves. Forty embolization cases were engaged in this study, and 27 cases were fully reproduced in the simulation setting (69.23%). The simulation success was significantly lower in pulmonary embolizations (*p* = 0.031) and significantly higher in soft tissue (*p* = 0.032) and coil embolizations (*p* = 0.045). The overall simulation success was 7.8 out of 10 available points.

**Conclusions:**

Using stereolithography 3D printing in a standardized simulation concept, endovascular embolization techniques for treating acute internal hemorrhages in the chest and abdomen can be simulated and trained based on the patient-specific anatomy in a majority of the cases and at a broad spectrum of different causes.

**Supplementary Information:**

The online version contains supplementary material available at 10.1186/s13244-022-01206-7.

## Key points

- Stereolithography (SLA) 3D printing enables case-based simulation and training of acute arterial embolizations.
- Simulations can be performed in thoracic and abdominal organs from a variety of different causes.
- Coils, particles and cyanoacrylate can be used as embolic agents according to the real-life procedure.
- Novel biological tissue mimicking resin can be used to print transparent and flexible vascular models.
- Patient-specific SLA 3D printing has the potential to improve patient safety and revolutionize IR education.

## Introduction

### Background

Minimally invasive endovascular embolization techniques are nowadays well established in treating acute arterial bleedings caused by trauma, tumors, surgical complications, excessive anticoagulation and a variety of other causes [1–3]. Therefore, the education and training of the next generation of specialized interventional radiologists are of special interest. The technique requires in-depth skills and knowledge of appropriate embolic agents and their delivery systems. Challenges therefore are, among others, to choose the right equipment for a specific problem, to find and locate the hemorrhage using digital subtraction angiography (DSA), to safely navigate the catheter and microcatheter to the artery of interest and to choose and correctly place the right kinds of embolic agents, which is often based on the physician’s experience [3]. The European Curriculum and Syllabus of the Cardiovascular and Interventional Radiological Society of Europe provides already detailed standards in teaching, yet the traditional apprenticeship model itself, where the trainee learns directly at the patient, depends on the individual skills of the teacher and potentially affects the patient’s safety [4, 5]. To overcome these issues, simulation training is now of growing interest in several medical and surgical fields. The related discipline Interventional Cardiology, for example, has already listed simulation trainings in their official curriculum as a learning method for catheter skills outside of the cath laboratory [6]. But also for the relatively young and considerably growing field of IR, simulation was already stated to have the potential to revolutionize and shorten the interventional skill training [7, 8].

Several methods and technologies have been described for endovascular simulation training in the recent past. McLeod et al., for example, introduced a perfused cadaveric aortic model for complex endovascular procedure training. But besides the realistic anatomical conditions of a cadaveric model, this approach requires great technical and financial efforts including a heart–lung machine, results in a radiation exposure for the trainee and has the major disadvantage of absence of pathologies [9]. In contrast, virtual reality (VR) allows training of interventional procedures with patient-specific anatomy and pathology. Using such case-based simulation concepts with VR, entire teams can be trained for the management of endovascular procedures [10]. Nevertheless, the technology still has many limitations, most importantly the lack of haptic feedback required for motor skill training [11]. The rapidly rising 3D printing technology, also known as additive manufacturing, offers a promising alternative to the limitations of cadaveric and VR training by enabling patient-specific simulations at relatively low costs and less technical efforts [11, 12]. 3D printing has already been described for endovascular simulations [13–16], but has not yet been investigated for embolization simulation of arterial bleedings in consecutive case studies.

### Objective

The objective of this study was to design and develop an adaptable endovascular simulation system for embolization training in patients with life-threatening traumatic and non-traumatic hemorrhages, integrating case-specific stereolithography (SLA) 3D printing into a standardized simulation concept. We evaluated the feasibility to simulate and technically replicate real-life endovascular embolization techniques in a consecutive case series over more than a year.

## Methods

### Patient selection, demographics and clinical data

We used our Picture Archiving and Communication System (PACS) system to find all cases of traumatic and non-traumatic hemorrhages treated with superselective arterial embolization at our institution from 1st of October 2018 until 31st of December 2019. The inclusion criteria were an embolization performed for acute bleeding and a pre-interventional planning CT in the arterial phase with axial reconstructions of 1 mm slice thickness for accurate 3D segmentation. Out of 87 embolizations, 40 cases were included in this retrospective study. The CT scans were performed with a 128-slice multidetector CT (Somatom Definition AS+, Siemens, Erlangen, Germany) using a standard protocol with a bolus of 120 mL non-ionic contrast media at an injection-rate of 3.5–5 mL/s. The arterial phase (CTA) was acquired at a delay of 30 s after injection. Standard demographic and clinical information was obtained to give a clinical overview of our study cohort. All patient identifiers were irreversibly removed and replaced by a random ID. This study has been approved by the local ethics committee (IRB-No.: 1004/2020).

### 3D printing with biological tissue mimicking resin

The CT scans in the arterial phase were imported to the open-source medical image processing software ImageJ (version 1.53, Laboratory for Optical and Computational Instrumentation, University of Wisconsin, USA) for segmentation [17]. The window-level tool was used for segmenting the inner lumen of the case-related arteries, and surface (STL) models were created using the 3D-Viewer tool with standard settings (display as: surface, color: white, threshold: 50, resampling factor: 2). The STL files were consecutively imported to the open-source 3D graphics software Blender (version 2.92, Blender Foundation, Amsterdam, The Netherlands) for post-processing [18]. Non-vascular structures were removed using the modeling tools and the select-linked tool, subdivision surface modifier to remove sharp edges (algorithm: catmull–clark, iterations: 1) and solidify modifier to generate the vessel walls with 1 mm wall thickness (mode: complex, thickness mode: fixed, boundary shape: none). The digital vascular models were printed with a biological tissue mimicking resin with flexible and transparent material properties (Flexible 80A, Formlabs, Massachusetts, USA) using a SLA 3D printer (Form 3, Formlabs, Massachusetts, USA) with a layer thickness of 0.1 mm and automatic generation of support structures (density: 0.8, touching point size: 0.4 mm, internal supports: deactivated). For post-processing, the models were cleaned automatically for 10 min in isopropanol (Form Wash, Formlabs), flushed with isopropanol injection by hand, uncaged from support structures and cured in ultraviolet light for 5 mins with 40 °C (Form Cure, Formlabs).

### Simulator concept and design

**Fig. 1:**
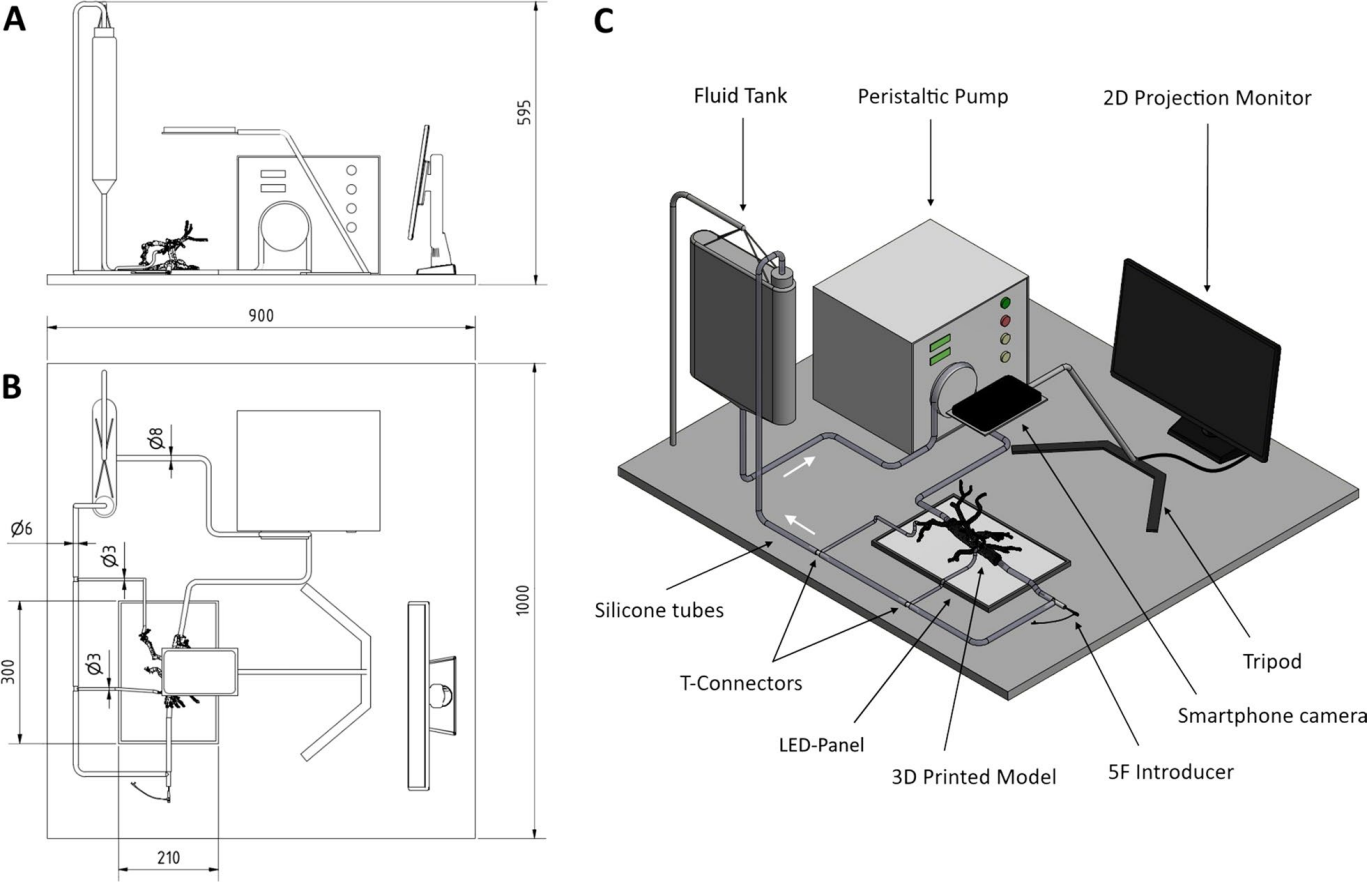
Technical illustration of the adaptable endovascular simulation setup. A patient-specific 3D printed vascular model is connected via silicone tubes to a peristaltic pump to establish a closed pulsatile circulatory system. The 2D projection in angiography is simulated with a smartphone camera on a tripod projected on a monitor. **A** Lateral view, **B** top view, **C** 3D visualization. Units: mm

An adaptable and cost-effective endovascular simulator was designed to enable case-based simulations with the patient-specific 3D printed vascular models. The concept involved an idle peristaltic pump for instillation of contrast media (Guerbet KMP 2000, Villepinte, France) with its included fluid tank, silicone tubes with diameters of 8 mm, 6 mm, 4 mm and 2 mm, compatible connectors for the vascular models (chapter 2.4), a LED panel and tripod for a smartphone camera with a monitor for 2D projection. The fluid tank and peristaltic pump were connected to the vascular models via the silicone tubes and their fitting connectors to establish a closed circulation. A blood-mimicking fluid, suitable for vascular phantoms, was mixed based on 47% water, 37% glycerol and 16% sodium iodide salt (each by weight) as proposed by Yousif et al [19]. A light-emitting diode (LED) panel was used for background lighting of the vascular model to improve the transparency and visibility of the endovascular devices during the simulation. The smartphone camera was attached to the tripod and connected to a monitor to simulate the 2D projection in angiography. A T-shaped connector and silicone tubes were used to place a 5-French sheath introducer to access the vascular model. Figure 1 illustrates and summarizes the simulator concept and design in a technical drawing.

### Pulsatile circulation and “Model-To-Pump” connection system

**Fig. 2:**
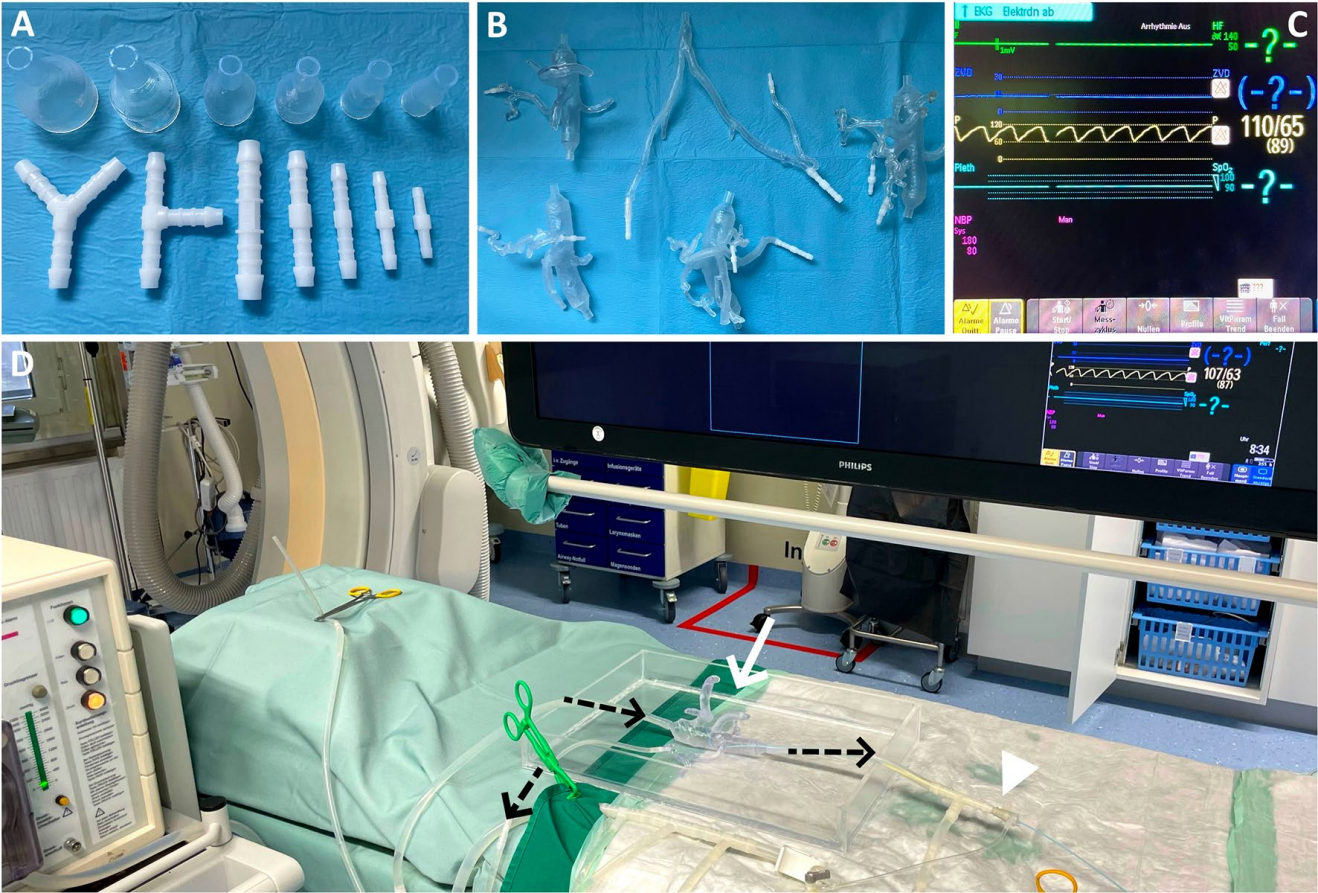
Connection system and pressure measurements. **A** Straight, Y- and T-shaped tube connectors (white) and the solid 3D printed funnel-shaped connectors in different dimensions. **B** A set of vascular models with chemically bonded connectors. **C** Physiological arterial pressure gradients of 110–80 mmHg were established in the 3D printed vascular models using the peristaltic pump. **D** Intravascular pressure measurements of the adaptable endovascular simulation system in the angiography suite. White arrow: 3D printed vascular model with introduced catheter. White arrowhead: 5-French introducer. Black arrows: Flow direction of the circulatory system

A peristaltic pump works by a rotor with two or three rollers compressing a flexible silicone tube externally. This, on the one hand, enables fluids to circulate through a closed system without contact to the pump and its technical components, which is essential for applying embolic agents like n-butyl cyanoacrylate without destroying the pump. And, on the other hand, it creates a pulsatile flow curve, which can be modified by the flow rate and the outflow resistance by using clamps to establish physiological pressure gradients. Pressure measurements were performed in the angiography suite and a gradient of 110–65 mmHg was reached inside the 3D printed vasculature, demonstrated in Fig. 2. The flexible, biological tissue mimicking material facilitated actual pulsating vascular models. To connect them to the peristaltic pump, an adjustable connection system was used. Standard straight, *T*- and *Y*-shape tube connectors in the dimensions of 3–8 mm (with 1 mm increment) were used for the connection of the 3D printed arteries to the silicone tubes and to connect and combine silicone tubes of different diameters. Funnel-shaped connectors were 3D printed with rigid Clear Resin (Formlabs, USA) in the dimensions of 18–32 mm maximum diameter (2 mm increment) and 8 mm minimum diameter, to connect a 3D printed aorta to the 8 mm silicone tubes of the peristaltic pump. To prevent fluid leakages at untight connections, the connectors were adhered to the 3D printed vessels using chemical bonding with liquid resin and a UV laser light pen (405 nm wavelength). Figure 2 also demonstrates the different standard tube connectors, the 3D printed connectors and a set of vascular models with chemically bonded connectors.

### Embolization equipment

The endovascular techniques were simulated and replicated using standard angiography equipment according to the real-life procedure. Catheters (4–5 French/Fr., Cordis, Florida, USA and Terumo, Tokyo, Japan), guidewires (0.035”, Terumo, Tokyo, Japan), microcatheters (2.5 Fr., Cook Medical LLC, Bloomington, USA) and microguidewires (0.014′′ and 0.016′′, Boston Scientific, Massachusetts, USA) were used to navigate through the 3D printed vasculature to the target artery of each embolization procedure. Microcoil embolizations were performed with expired detachable training coils in dimensions of 2–8 mm diameter (INTERLOCK™-18 Detachable, Boston Scientific, Massachusetts, USA), depending on each procedure. Microparticle embolizations were simulated using Contour PVA particles in the dimensions of 250–355 µm (Boston Scientific, Massachusetts, USA) mixed into an orange colored saline solution, to make the embolic agent visible through the transparent 3D printed models. The blue colored Histoacryl (Braun, Melsungen, Germany) and Lipiodol (Guerbet, Villepinte, France) with preceding glucose saline solution injection were used for n-butyl cyanoacrylate embolization simulations because of its visibility in the transparent 3D printed models. Disappearing ink was used to replace contrast media injections in this closed circulatory simulation system, which quickly resolves and keeps the blood-mimicking fluid transparent.

### Simulation and evaluation

The real-life cases were performed by five different interventional radiologists, all retrospective simulations were performed by the same interventional radiologist. The feasibility of reproducing and practicing real-life embolizations retrospectively in this simulation concept was investigated using a standardized evaluation model. This model involved the main steps of 3D printing, simulation, catheterization and embolization where each step was rated with 1 point with a maximum of 10-points for each case. A total of 5 points were given for the procedural steps from catheterization to embolization. One case was not applicable for this evaluation model because it involved a covered stent to manage a hepatic artery bleeding without the use of a microcatheter-system. The whole evaluation model is summarized in Table 1.

**Table 1:** Evaluation model

Evaluation step	Points (0–10)
1. Visibility of the bleeding artery on the CT scan	1 Point (10%)
2. Segmentation	1 Point (10%)
*3D printing*	
3. Main artery printed	1 Point (10%)
4. Procedure-relevant artery printed	1 Point (10%)
*Simulation setup*	
5. Connection established	1 Point (10%)
*Catheterization*	
6. Guidewire advanced to the main artery	1 Point (10%)
7. Stable main catheter position in the main artery	1 Point (10%)
8. Microwire advanced to the bleeding artery	1 Point (10%)
9. Stable microcatheter position in the bleeding artery	1 Point (10%)
*Embolization*	
10. Application of the procedure-related embolic agents	1 Point (10%)
Total	0–10 Points (0-100%)

The technical evaluation model uses a 10-point grading scale to rate the success of each procedural simulation. It involves the main steps of 3D printing, simulation setup, catheterization and embolization

### Statistics

Data were checked for consistency and normality. Due to deviation from normality, bootstrap ANOVA were used to test for global effects concerning simulation success based on the evaluation model demonstrated in Table 1. Randomization tests were used for planned comparisons to analyze simulation success. Spearman's correlation coefficients were computed. All reported tests were two-sided, and *p*-values < 0.05 were considered statistically significant. All statistical analyses in this report were performed by use of NCSS (NCSS 10, NCSS, LLC. Kaysville, UT), STATISTICA 13 (Hill, T. and Lewicki, P. Statistics: Methods and Applications. StatSoft, Tulsa, OK) and RStudio Team (2020, RStudio: Integrated Development for R. RStudio, PBC, Boston, MA) [20].

## Results

### Demographics and clinical data

In total, 40 patients matched our criteria and were included in the final investigation. The study cohort was divided into subgroups of affected organs, bleeding causes and the different kinds of embolic agents. Most bleedings were treated with microcoils (*n* = 19), microparticles (*n* = 9) or microcoils and -particles in combination (*n* = 7). Four cases were performed with n-butyl cyanoacrylate. One patient was treated with a covered stent in case of a hepatic artery bleeding. All patients were analyzed for baseline demographic, clinical and interventional parameters and compared to the simulation success, summarized in Table 2. A significant correlation was found for the number of coils used in the intervention and the simulation success (*r* = 0.49, *p* = 0.001). All other baseline characteristics and interventional parameters were statistically not significant.

**Table 2:** Demographic and clinical overview and the comparison to the simulation success

Baseline characteristics	Mean ± SD/number (%)	Spearman correlation	*p*
Age (years)	64.1 ± 18.4	0.09	0.57
BMI (kg/m^2^)	25.1 ± 5.7	0.21	0.21
BSA (m^2^)	1.8 ± 0.2	0.00	0.99
Hemoglobin (g/dl)	9.2 ± 2.1	0.07	0.68
Leukocytes (G/l)	11.6 ± 5.4	0.18	0.27
Thrombocytes (G/l)	297.4 ± 187.8	0.27	0.11
CRP (mg/dl)	5.3 ± 5.6	0.05	0.78
Creatinine (mg/dl)	1.3 ± 1.5	0.14	0.41
TSH (mU/l)	2.4 ± 2.2	0.24	0.14
Male gender	24 (60 %)	n/a	0.42
Coronary artery disease	6 (15 %)	n/a	0.43
Myocardial infarction	4 (10 %)	n/a	0.92
Arterial hypertension	22 (55 %)	n/a	0.22
Diabetes mellitus	9 (22.5 %)	n/a	0.54
History of stroke	8 (20 %)	n/a	0.70
Renal failure	8 (20 %)	n/a	0.21
Smoking	13 (32.5 %)	n/a	0.30
Alcohol	4 (10 %)	n/a	0.16
*Interventional parameters*			
Contrast agent (ml)	78.4 ± 28	− 0.02	0.92
Dose area product (mGy·cm^2^)	173.1 ± 186.2	0.27	0.11
Fluoroscopic time (min.)	25.1 ± 15.4	0.02	0.89
Number of coils	4.7 ± 4.6	0.49	0.001 *

Baseline characteristics including patient history and clinical data as well as interventional parameters of all patients included in this study. The laboratory data corresponds to the values directly before the embolization procedure. * = statistically significant

### 3D printed vascular models

**Fig. 3:**
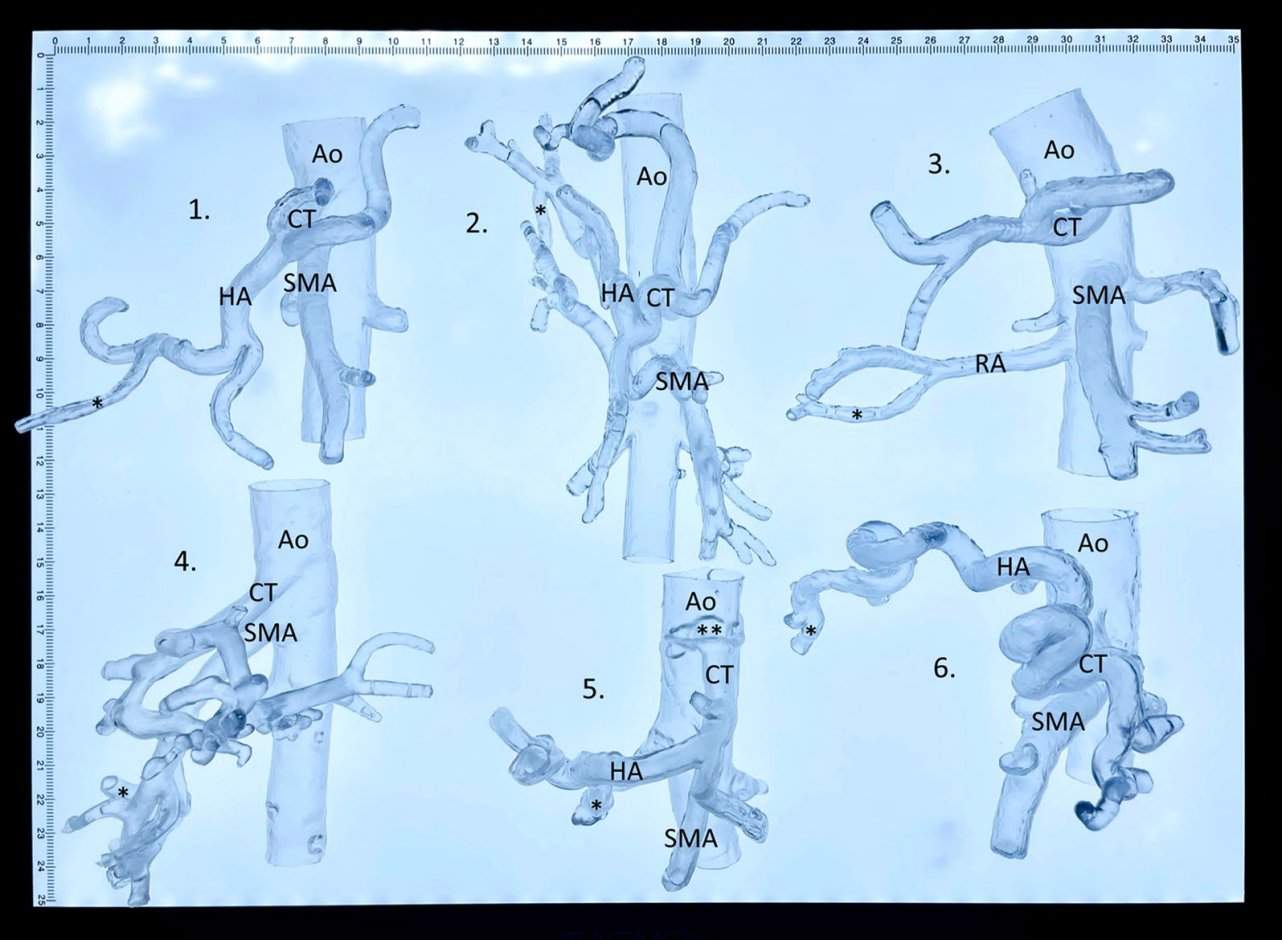
3D printed vascular models. A set of 3D printed visceral vascular models with patient-specific anatomy based on preprocedural computed tomography scans to demonstrate the vascular dimensions and the level of details for procedure simulations. 1. Spontaneous bleeding from ruptured cyst in polycystic liver disease, 2. spontaneous hepatic tumor bleeding, 3. post-surgery kidney bleeding, 4. pancreas tumor bleeding from the inferior pancreaticoduodenal artery, 5. gastroduodenal artery bleeding from duodenal ulcer, and 6. liver metastasis bleeding in esophageal cancer. Ao = aorta, CT = celiac trunk, SMA = superior mesenteric artery, HA = hepatic artery, RA = renal artery, * = procedure-relevant artery, ** = celiac trunk stenosis from an atherosclerotic plaque

Thirty-seven vascular models were segmented and printed using the open-source software and the biological tissue mimicking flexible resin (92.5%). In three cases the segmentation was not possible because the bleeding artery was not visible on the CT scan (7.5%). One print failed because of repeated model rupture during the 3D printing process. In the technically successful prints, the procedure-relevant artery was not included in 7 cases (17.5%), because the artery was not visible in the CT scan (3 cases; 7.5%), or because the segmentation of the procedure-relevant artery failed (4 cases; 10%). Consecutively, a total of 32 models was technically successfully printed including its procedure-relevant artery (80%). A set of patient-specific 3D printed vascular models are demonstrated in Fig. 3. Additional file 1 shows the flexible and transparent material behavior of the 3D printed models.

### Simulation and evaluation

**Fig. 4:**
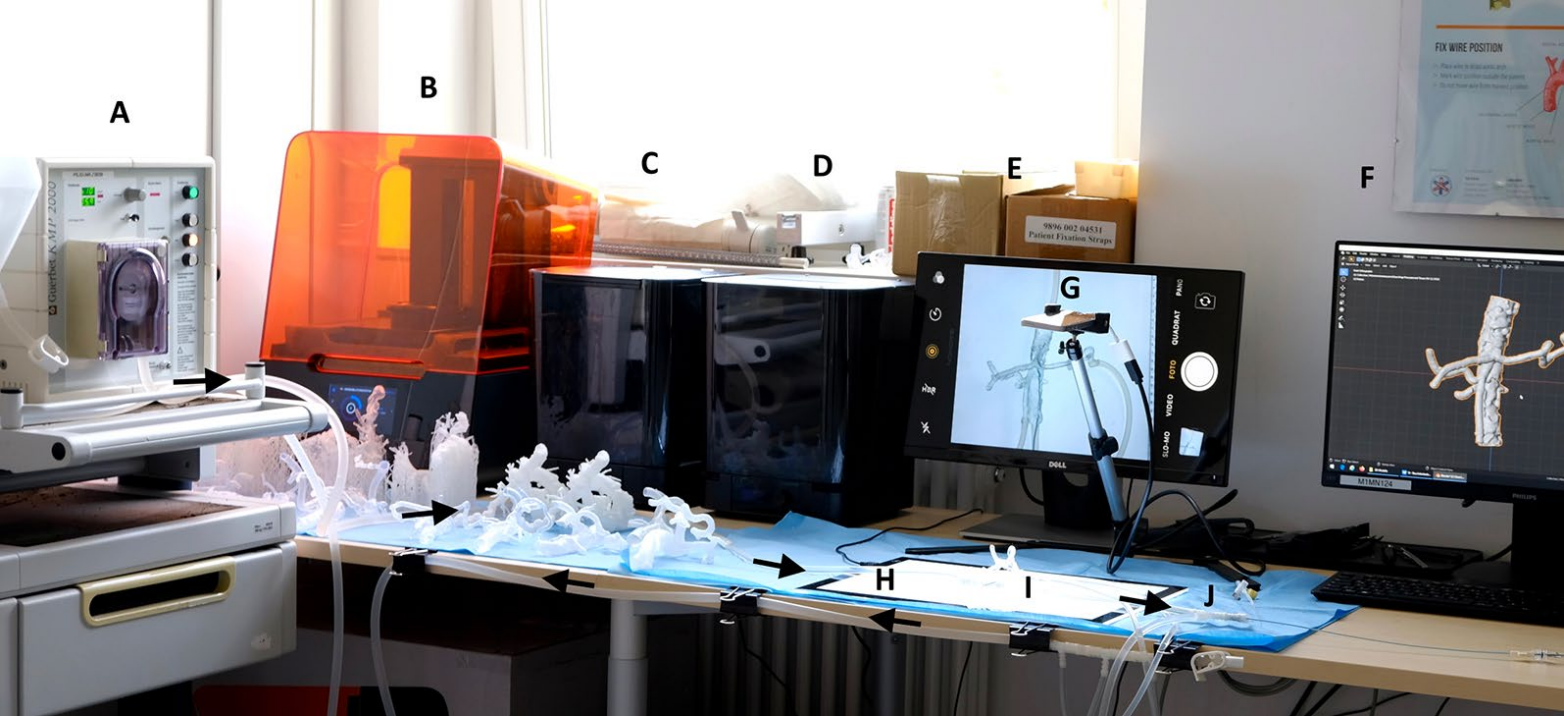
The 3D printing and simulation setup. This figure illustrates the concept of an in-house 3D print and simulation laboratory. **A** peristaltic pump, **B** SLA 3D printer, **C** Isopropyl-alcohol tank for cleaning of the 3D prints, **D** ultraviolet curing lamp, **E** monitor for 2D projection, **F** computer for segmentation, **G** smartphone camera on a tripod, **H** LED panel, **I** 3D printed vascular model, **J** 5-French introducer. Black arrows demonstrate the flow direction of the circulatory system

**Fig. 5:**
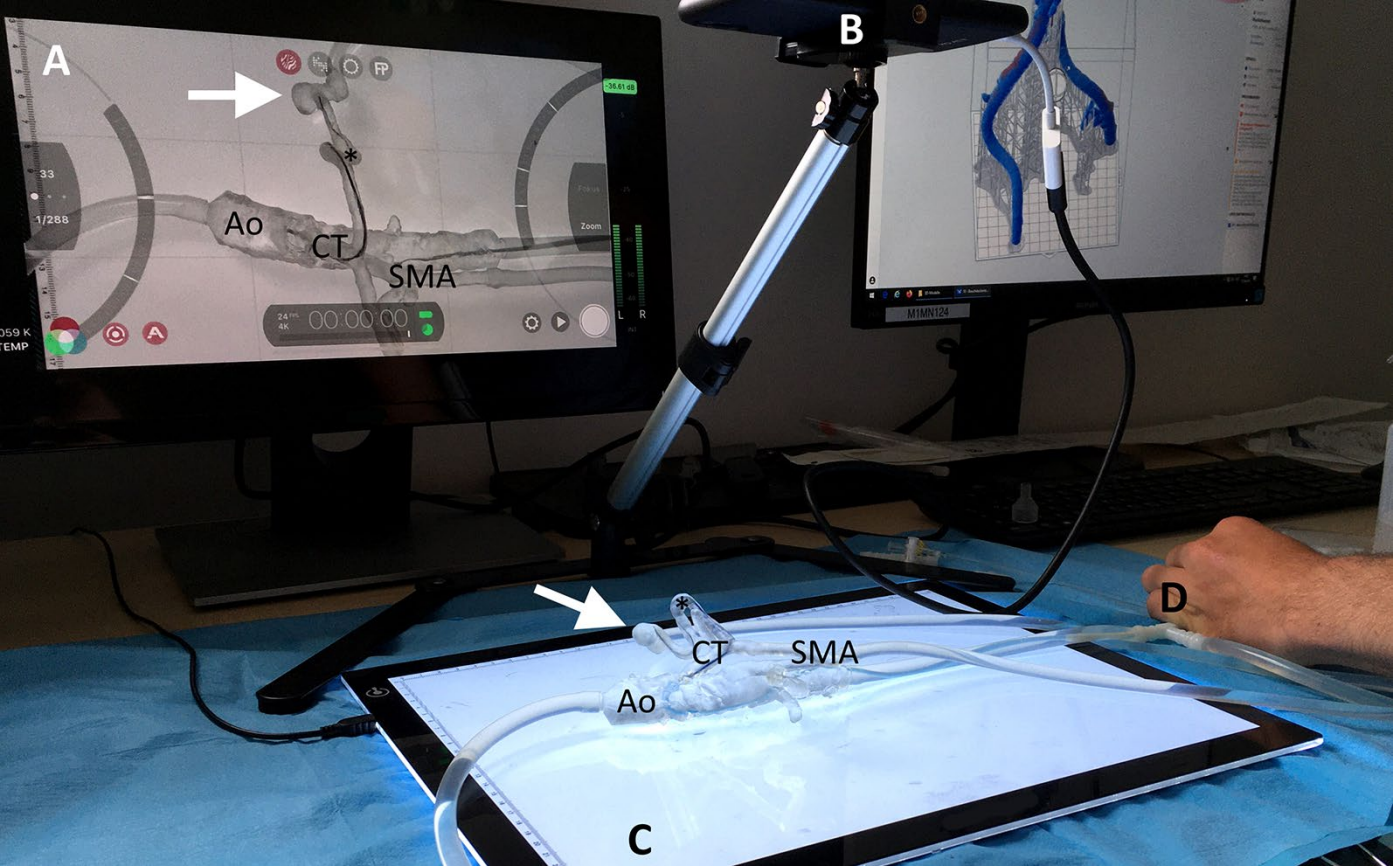
Procedural simulation. A trainee practicing the catheterization of a splenic artery with pseudoaneurysm (white arrow). A 5-French Cobra C2 catheter is placed in the celiac trunk and a microcatheter with guidewire is advanced to the splenic artery. **A** 2D projection of the simulation model, **B** Smartphone camera on a tripod, **C** LED panel for improved visibility, **D** 5-French introducer sheath, Ao = aorta, CT = celiac trunk, SMA = superior mesenteric artery, * = splenic artery

**Fig. 6:**
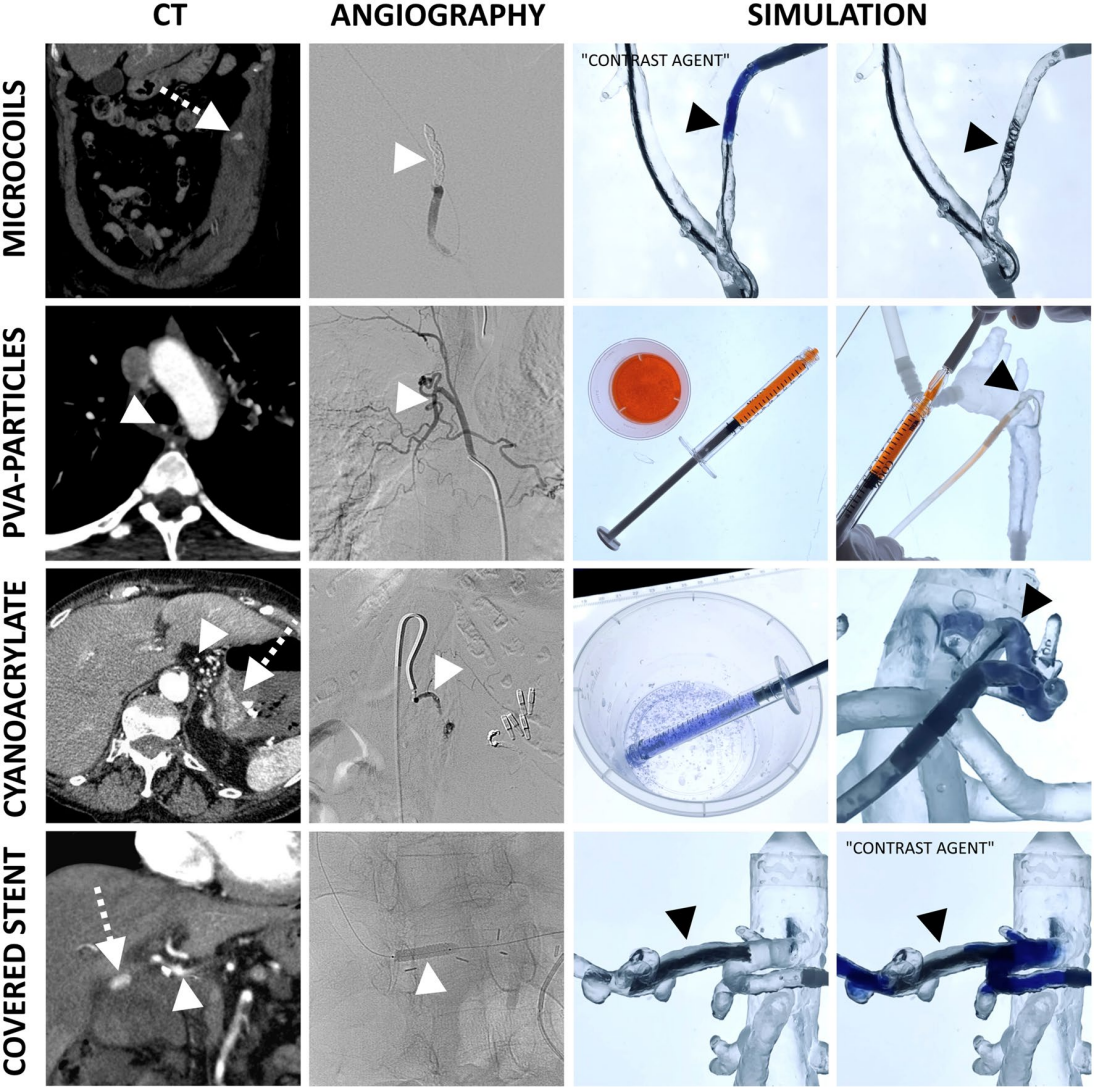
Embolic agents. This figure demonstrates the different embolic agents used in this simulation setting. White arrows with dotted lines = active bleeding. White and black arrowheads = procedure-relevant artery. Blue colored disappearing ink was used to replace contrast media, which quickly resolves within seconds to become invisible in this closed circulatory system. PVA particles were mixed with an orange colored saline solution for better visibility. For the same reason, Histoacryl (light blue) was used for n-butyl cyanoacrylate embolizations

**Fig. 7:**
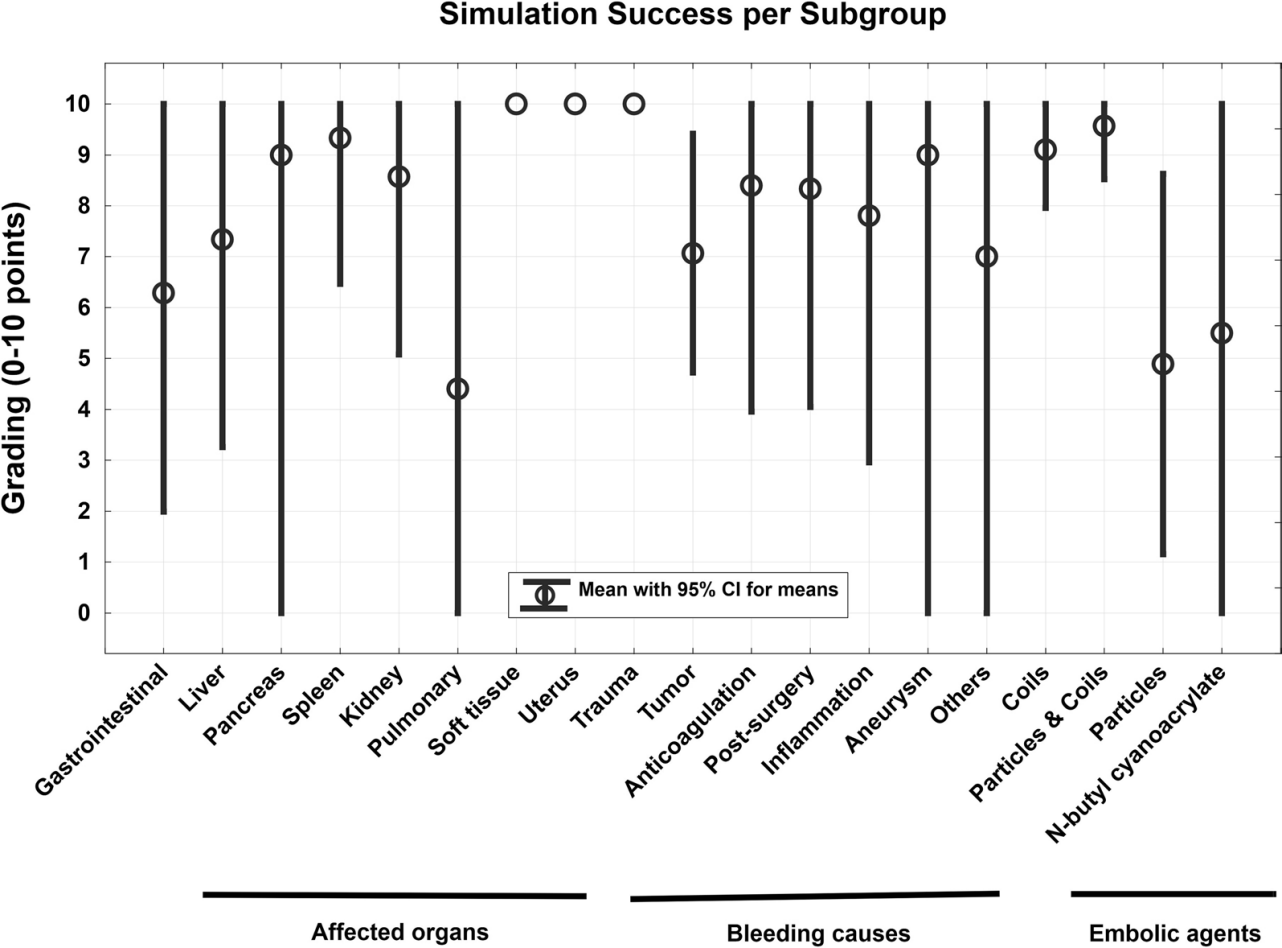
Box plots for simulation success. Simulation success is listed in groups of affected organs, bleeding causes and embolic agents. The simulation success in the 10-point grading scale was significantly reduced in pulmonary embolization (*p* = 0.031) and significantly increased in soft tissue (*p* = 0.032) and coil embolizations (*p* = 0.045)

The simulation concept including the in-house 3D print laboratory is shown in Fig. 4. The case-specific vascular models were connected to the circulatory system and the embolization procedures were reproduced in accordance with the related real-world embolization procedure (see Fig. 5). The embolic agents used in this case series were successfully applied in the simulation setting (see Fig. 6). The mean grading of all cases was 7.85 points. Twenty-seven of 39 cases were rated with the full amount of points and therefore completely reproducable (69.23%). The subgroup analysis revealed that simulations with microcoils as embolic agent and cases with soft tissue as affected organ had a significantly higher rating in this evaluation model (*p* = 0.045 and *p* = 0.032). In cases of pulmonary embolizations, the simulation success was significantly reduced with a mean success of 4.4 ± 4.6 (*p* = 0.031). For all other subgroups no significant differences were observed as demonstrated in Table 3. Figure 7 visualizes the simulation success of all subgroups by means of box plots. In the case with a covered stent as embolic agent, the stent was placed, but not in an ideal position in the simulation setting because it was not possible to fully advance the stent graft.

**Table 3:** Subgroup analysis

Subgroups	Cases (#)	Average rating ± SD	*p*
*Affected organ/system*			
Liver	7	7.3 ± 3.5	0.72
Pancreas	2	9 ± 1	0.60
Spleen	3	9.3 ± 0.9	0.59
Kidney	8	8.6 ± 3.5	0.59
Gastrointestinal	7	6.3 ± 4.3	0.28
Pulmonary	5	4.4 ± 4.6	0.031 *↓
Uterus	1	10 ± 0	0.90
Soft tissue	7	10 ± 0	0.032 *↑
*Cause of hemorrhage*			
Aneurysm	2	9 ± 1	0.92
Anticoagulation	5	8.4 ± 3.2	0.62
Inflammation	5	7.8 ± 3.5	1.00
Post-surgical	7	8.3 ± 3.7	0.80
Trauma	2	10 ± 0	0.45
Tumor	16	7.1 ± 4.1	0.30
Others*	3	7 ± 4.2	0.94
*Embolic agent*			
Microcoils	19	9.1 ± 2.3	0.045 *↑
Microparticles	9	4.9 ± 4.6	0.10
Microparticles and coils	7	9.6 ± 1	0.25
N-butyl cyanoacrylate	4	5.5 ± 4.6	0.16
Covered stent	1	n/a	n/a
Total	40	7.8	

This table demonstrates the simulation success in categories of three subgroups: affected organ, cause of hemorrhage and embolic agent. * marks significant differences

### Simulation video

How this endovascular simulation concept works in reality is demonstrated in Additional file 2. The aortic bifurcation is crossed to treat a spontaneous inferior epigastric artery bleeding from anticoagulation using microcoils in the dimensions of 3 mm diameter. A kidney tumor embolization with microparticles is simulated using the orange colored saline solution with 250–355 μm particles. A n-butyl cyanoacrylate embolization is simulated in a case of a bleeding gastric ulcer, where full embolization is reached also in the simulation setting. Finally, a covered stent is placed in the 3D printed hepatic artery to treat an acute bleeding from surgery. The video further demonstrates the transparent and flexible behavior of the biological tissue mimicking 3D printing material as well as the pulsating effect from the peristaltic pump.

### Error analysis

Twelve of 39 cases were not fully reproducible in the simulation and evaluation (30.77%). One case failed because of a 3D print error even after multiple attempts and 4 cases failed because the segmentation was not possible as mentioned in Section 3.2. In 2 cases the microwire advancement to the procedure-relevant artery was not possible and in 2 other cases a stable microcatheter position could not be established (10.26 % in total). No error occurred because of a failed connection of the vascular model to the simulator. Also no error occurred in placement of the main guidewire and catheter. The different kinds of errors are listed in Table 4.

**Table 4:** Error analysis

Error analysis	Amount	Percent
Lack of visibility on CT (CT error)	3	7.69
Segmentation error	4	10.26
3D Printing error: Main artery	1	2.56
3D Printing error: Bleeding artery	0	0.00
Connection to simulator failed	0	0.00
Guidewire placement failed	0	0.00
Main catheter placement failed	0	0.00
Microwire placement failed	2	5.13
Microcatheter placement failed	2	5.13
Embolization failed	0	0.00
Overall failure (0–9 points/0–90%)	12	30.77
Overall success (10 points/100%)	27	69.23

This table summarizes the technical and procedural errors that occurred in this case series

## Discussion

### Principal findings

Interventional radiology (IR) today plays a crucial role in the management of life-threatening acute hemorrhages in a variety of causes including trauma, surgery complications, tumors, adverse drug effects and more [1–3]. In this interventional endovascular approach, the operator is able to locate and precisely navigate to the bleeding artery using guidewires, catheters and superselective microcatheters to finally stop the bleeding using various embolic agents at high technical success rates of 50–100% [21]. For trauma patients with arterial bleedings for example, a reduction of the devastating mortality rates from 26 to 10% was reported when comparing open surgery to minimally invasive IR embolization [22]. Furthermore, the IR procedures are less invasive than surgery which results in faster recovery times, reduced length of stay in intensive care units and earlier discharges [23]. The diagnosis of acute hemorrhage is usually made by a preceding diagnostic computed tomography scan (CT), followed by the fast and effective IR treatment [24, 25].

The apprenticeship model has been used since generations for education and training of technical skills in IR and other fields. In this traditional model, the unexperienced trainee learns and practices surgical or interventional techniques from an experienced specialist in the operating room directly at the patient [26]. This leads to increasing concerns about patient safety and quality of care [27], but also comes with further challenges including declining training opportunities due to reduced working hours and changes in healthcare systems [28]. In addition to that comes the problem, that acute cases of arterial bleedings cannot be planned, which is why interventionalists in training have to be in the right place at the right time to learn these procedures. To overcome these issues, simulation concepts are of increasing interest as an adjunct to the apprenticeship model, to move the initial training outside the operating room to a safe environment for motor skill acquisition [29].

In a preceding study we have proven the feasibility and technical accuracy of vascular 3D printing with a novel biological tissue mimicking resin [30]. In this consecutive study, we developed a simulation concept based on such biological tissue mimicking vascular models. Endovascular embolization techniques were simulated in a consecutive case series of acute arterial bleedings. A peristaltic pump, a “model-to-pump” connection system and blood-mimicking fluid were utilized to establish a pulsatile circulation with verified almost physiological pressure gradients. Microcoils, PVA particles and n-butyl cyanoacrylate were used as embolic agents in a variety of different affected organs and bleeding causes. Over more than a year, all acute embolizations with matching inclusion criteria were included in this investigation and rated for each technical and procedural step with an overall success rate of 69.23%. In these cases, the endovascular embolizations were successfully replicated in the simulation setting according to their real-life procedures and to the patient-specific anatomy (10 out of 10 points/100%).

In 30.77% of the cases, the procedures were not fully reproducible (< 10 points/< 100%), which was in the majority of the cases caused by a segmentation error (10.26%). This error occurred, when the bleeding artery had a reduced contrast or, in cases of bronchial artery bleedings, had a close-by course to aorta, which lead to a fusion of the procedure-relevant artery with the aorta. Whether this issue was caused by our segmentation method or if this is a general problem in segmentation of bronchial arteries remains unclear at this stage and has to be further investigated in future studies. However, the second most frequent error was a lack of visibility of the procedure-relevant artery on the CT scan (7.69%), which was not caused by segmentation, 3D printing or the simulation setup and is therefore a simulation-independent error. In four cases the microwire or microcatheter advancement to the bleeding artery failed (10.26%). Due to technical reasons of 3D printing layer-by-layer and the resin material itself, the inner surface of 3D printed vessels is surely not as smooth as a real vessel and this aspect probably made superselective catheterization more difficult in some cases. Only in one case the 3D printing itself failed (2.56%), caused by extensive vascular kinking.

The subgroup analysis revealed a significantly higher simulation success in cases with microcoils and soft tissue bleedings. Furthermore, a significant correlation was found between the number of coils used in the embolization procedure and the simulation success. Microcoils were preferred as embolic agent when the bleeding artery was large enough to place a coil. It is highly suggestive that the larger vessels caused a reduction of errors in the cases with microcoils and that the segmentations were easier in the soft tissue bleedings because of a less complex anatomy (i.e., inferior epigastric artery) and fewer anatomical surroundings. A significantly reduced simulation success was noted in pulmonary bleedings, which was mainly caused by lack of detail on the CT scan and the segmentation errors mentioned above. In one case which was treated with a covered stent, it was technically more challenging to advance this stent to the hepatic artery in comparison to the real-life procedure, which resulted in partial misplacement proximal to its ideal position. We cannot exclude that this might be a general issue in simulations with covered stents in 3D printed models with this resin; also, it remains unclear at this stage if this problem persists in other 3D printing technologies or materials, which demands further investigations. Further research is also necessary to evaluate the potential of comparing angiography equipment like different catheters, wire shapes or embolic agents in the same case. No demographic or clinical confounders were observed.

### Limitations

There are clearly some limitations of this study and simulation concept. In contrast to traditional animal models for example, full embolization until stasis cannot be reached with coils and microparticles as coils need blood clotting and microvasculature is neither visible on the CT nor printable with standard SLA 3D printers. Yet, the technical applications of microcoils and microparticles with the potential risks of misplacement, backflow and reflux can be simulated. Full embolization, however, was only reached with n-butyl cyanoacrylate. 3D printed microfluidic devices could be a possible solution for this problem considering microparticle embolizations, which should be investigated in future studies. The peristaltic pump in this concept was technically appropriate for this simulation setting with verified pressure gradients of 110 to 65 mmHg, yet not a true physiological flow curve was established. More realistic flow curves might be generated by pulsatile heart–lung machines or specific cardiovascular simulation pumps, but at higher costs. Also, commercial segmentation software might be useful, however, this study was focused on standard components and open-source software to make such simulations easier affordable and more accessible. Most importantly, 3D printing for patient-specific procedure simulations requires not only technical skills, but also in-depth knowledge of anatomy, multimodality imaging analysis and multidisciplinary clinical understanding. Experienced interventional radiologists are necessary to prevent inappropriate gamification of interventional procedures and instead use this technology in a professional educational context.

## Conclusions

The presented endovascular simulation concept facilitates the training of emergency embolization procedures in acute thoracic and abdominal arterial bleedings. The procedures can be technically reproduced and trained according to the patient-specific anatomy in a majority of the cases and at a broad spectrum of different causes. Such concepts have the potential to improve the patient safety by moving the initial hands-on training of emergency procedures off the patient and outside the IR cath laboratory.

## Supplementary Information

**Additional file 1**: Biological tissue mimicking resin. A novel flexible and transparent 3D printing material (Flexible 80A, Formlabs, USA) with biological tissue mimicking characteristics was used for 3D printing of the vascular models with SLA technology. This video demonstrates the flexible and transparent material properties of a 3D printed vascular model.

**Additional file 2**: Embolization simulation. This video shows different catheterization steps and cases including an iliac crossover maneuver as well as the application of three kinds of embolic agents: microcoils, microparticles and n-butyl cyanoacrylate. One case of hepatic artery bleeding in this series was treated and simulated with a covered stent.

## Data Availability

The datasets used and/or analyzed during the current study are available from the corresponding author on reasonable request.

## Abbreviations

CT: Computed tomography
DSA: Digital subtraction angiography
IR: Interventional radiology
PVA: Polyvinyl alcohol
SLA: Stereolithography
STL: Stereolithography file format
VR: Virtual reality
